# Errors in RNA-Seq quantification affect genes of relevance to human disease

**DOI:** 10.1186/s13059-015-0734-x

**Published:** 2015-09-03

**Authors:** Christelle Robert, Mick Watson

**Affiliations:** The Roslin Institute and Royal (Dick) School of Veterinary studies, University of Edinburgh, Easter Bush, EH25 9RG UK; Edinburgh Genomics, The Roslin Institute, University of Edinburgh, Easter Bush, EH25 9RG UK

## Abstract

**Background:**

RNA-Seq has emerged as the standard for measuring gene expression and is an important technique often used in studies of human disease. Gene expression quantification involves comparison of the sequenced reads to a known genomic or transcriptomic reference. The accuracy of that quantification relies on there being enough unique information in the reads to enable bioinformatics tools to accurately assign the reads to the correct gene.

**Results:**

We apply 12 common methods to estimate gene expression from RNA-Seq data and show that there are hundreds of genes whose expression is underestimated by one or more of those methods. Many of these genes have been implicated in human disease, and we describe their roles. We go on to propose a two-stage analysis of RNA-Seq data in which multi-mapped or ambiguous reads can instead be uniquely assigned to groups of genes. We apply this method to a recently published mouse cancer study, and demonstrate that we can extract relevant biological signal from data that would otherwise have been discarded.

**Conclusions:**

For hundreds of genes in the human genome, RNA-Seq is unable to measure expression accurately. These genes are enriched for gene families, and many of them have been implicated in human disease. We show that it is possible to use data that may otherwise have been discarded to measure group-level expression, and that such data contains biologically relevant information.

**Electronic supplementary material:**

The online version of this article (doi:10.1186/s13059-015-0734-x) contains supplementary material, which is available to authorized users.

## Background

Transcriptomics is an important approach that has helped researchers understand the molecular basis of disease in a range of species. Whilst for many years microarrays were the tool of choice, RNA-Seq [1] has now emerged as the standard method for analysing the transcriptome, contributing to thousands of publications in the biomedical literature. High throughput, second generation sequencers routinely output several hundred million reads at very low cost, and RNA-Seq is the application of those sequencers to RNA that has undergone conversion to cDNA. The result is that researchers can cheaply generate tens of millions of reads per sample, allowing them to both measure expression and reconstruct splice isoforms [2]. RNA-Seq is now fundamental to many large functional annotation projects, such as ENCODE [3], a large multi-national effort to define functional elements in the human genome.

There are many existing bioinformatics approaches to RNA-Seq quantification — the conversion of raw sequencing reads into estimates of gene expression. The most popular approach involves aligning the reads to a reference genome (or transcriptome) using a spliced aligner such as TopHat [4] or STAR [5]. The alignment step is very computationally intensive, with each sample taking many hours, depending on tool and parameter choices. The result is that each read (or fragment) is assigned zero, one or many putative locations within the reference sequence. Reads that map in multiple locations are described as multi-mapped; in addition, any given mapping location may overlap with multiple genes in the annotation, and these are described as ambiguously mapped reads. How the multi-mapped/ambiguous reads are handled and reported is dependent on the software chosen, and is a major source of error in RNA-Seq quantification. Given a set of alignments, additional tools are needed to assign reads to genes to quantify gene expression. Some use simple counting techniques against a known annotation [6], whereas others simultaneously construct transcripts and model-based estimates of gene expression [2].

Short-read alignment is a complex problem, and in RNA-Seq this is further compounded by gene families. Having many members with identical or close-to-identical sequences, gene families are often enriched for multi-mapped reads; therefore, the results of RNA-Seq quantification depend on the choice of aligner, the choice of the reference, a huge array of parameters, and algorithmic details relating to how multi-mapped reads are handled and reported. The choice of quantification tool also has a huge effect, as these also differ in the way they handle aligned data and multi-mapped/ambiguous reads.

Recently, Patro et al*.* [7] described a new method which builds an index of unique kmers within transcripts, and uses those to estimate gene expression directly from the raw reads. The algorithm reports a 25 times faster run time than other approaches, with equivalent accuracy. However, it is unable to discover novel transcript isoforms or splice junctions (a key benefit of RNA-Seq), and by relying on kmers, which are necessarily less than the read length, they are likely to suffer similar issues to those caused by multi-mapped reads.

At the heart of RNA-Seq is an assumption that the method produces reliable measurements of gene expression, and a recent paper has suggested that this may not be the case [8]. In this study we test biases introduced by the bioinformatics aspects of RNA-Seq quantification — that is, the conversion of raw sequencing reads into estimates of gene expression. We test a wide range of techniques and find systematic biases within each of them, resulting in severe underestimation or overestimation of gene expression in hundreds of genes, many of which have relevance to human disease. We go on to propose a two-stage RNA-Seq analysis that allows researchers to discover biological signal within data that may otherwise have been discarded.

## Results

### Simulation of reads from all protein-coding genes

We simulated 1000 perfect RNA-Seq read pairs from each of 19,654 protein-coding genes, and estimated their gene expression using 12 different methods (see “Materials and methods”; Table 1). Of 19,654 million read pairs, TopHat reported 850,613 fragments without a unique mapping (4.33 %) whereas STAR reported 583,308 (2.97 %).

**Table 1:** Method description

Method	Aligner	Quantification	Quantification notes
star.htseq. u	STAR 2.4.0	htseq-count (HTSeq 0.6.1)	-m union
star.htseq.ine	STAR 2.4.0	htseq-count (HTSeq 0.6.1)	-m intersection-strict
star.htseq. is	STAR 2.4.0	htseq-count (HTSeq 0.6.1)	-m intersection-nonempty
tophat.htseq. u	TopHat 2.0.12	htseq-count (HTSeq 0.6.1)	-m union
tophat.htseq.ine	TopHat 2.0.12	htseq-count (HTSeq 0.6.1)	-m intersection-strict
tophat.htseq. is	TopHat 2.0.12	htseq-count (HTSeq 0.6.1)	-m intersection-nonempty
star.cufflinks	STAR 2.4.0	Cufflinks 2.2.1	
star.cufflinks.mr	STAR 2.4.0	Cufflinks 2.2.1	--multi-read-correct
tophat.cufflinks	TopHat 2.0.12	Cufflinks 2.2.1	
tophat.cufflinks.mr	TopHat 2.0.12	Cufflinks 2.2.1	--multi-read-correct
sailfish	NA	Sailfish 0.6.3	quant.sf
sailfish	NA	Sailfish 0.6.3	quant_bias_corrected.sf

A description of the RNA-Seq alignment and quantification methods used. *NA* not applicable

Read counts and estimates of gene expression for the 12 methods and 19,654 genes can be seen in Additional file 1: Table S1. In total, 843 (4.31 %) genes were assigned a read count less than 100 by at least one of the methods, and 586 (2.98 %) were assigned a read count of zero, suggesting that those genes are completely undetectable by the method(s) in question. A total of 187 genes were assigned a read count greater than 1900 by at least one of the methods. The two lists (<100 or >1900) are not mutually exclusive, as some genes were underestimated by one method yet overestimated by another. In total, 958 genes were assigned vastly under- or overestimated read counts by at least one method.

When comparing expected with reported FPKM (fragments per kilobase per million; see “Materials and Methods”) values, Sailfish (*r* = 0.953), TopHat plus Cufflinks (*r* = 0.953) and STAR plus Cufflinks (*r* = 0.951) performed best, although the accuracy of the Cufflinks methods dropped to *r* = 0.899 and *r* = 0.907, respectively, once the --multi-read-correct parameter was chosen. The bias correction values from Sailfish performed terribly (*r* = 0.083), and we suspect some problem with the bias correction model on these data (Table 2).

**Table 2:** Method performance on global simulated data

Method	Description	Pearson correlation coefficient
Star.htseq. u	STAR, HTSeq (union)	0.78
Star.htseq.ine	STAR, HTSeq (intersection-nonempty)	0.88
Star.htseq. is	STAR, HTSeq (intersection-strict)	0.86
Tophat.htseq. u	TopHat2, HTSeq (union)	0.78
Tophat.htseq.ine	TopHat2, HTSeq (intersection-nonempty)	0.87
Tophat.htseq. is	TopHat2, HTSeq (intersection-strict)	0.86
Star.cufflinks	STAR, Cufflinks	0.95
Star.cufflinks.mr	STAR, Cufflinks (multi-read-correct)	0.91
Tophat.cufflinks	TopHat2, Cufflinks	0.95
Tophat.cufflinks.mr	TopHat2, Cufflinks (multi-read-correct)	0.90
Sailfish	Sailfish (RPKM)	0.95
Sailfish	Sailfish bias-corrected (RPKM)	0.08

Pearson correlation coefficients between FPKM from each method and the expected FPKM from simulated data for 19,654 human protein-coding genes. *RPKM* reads per kilobase per million (see “Materials and Methods”)

**Fig. 1:**
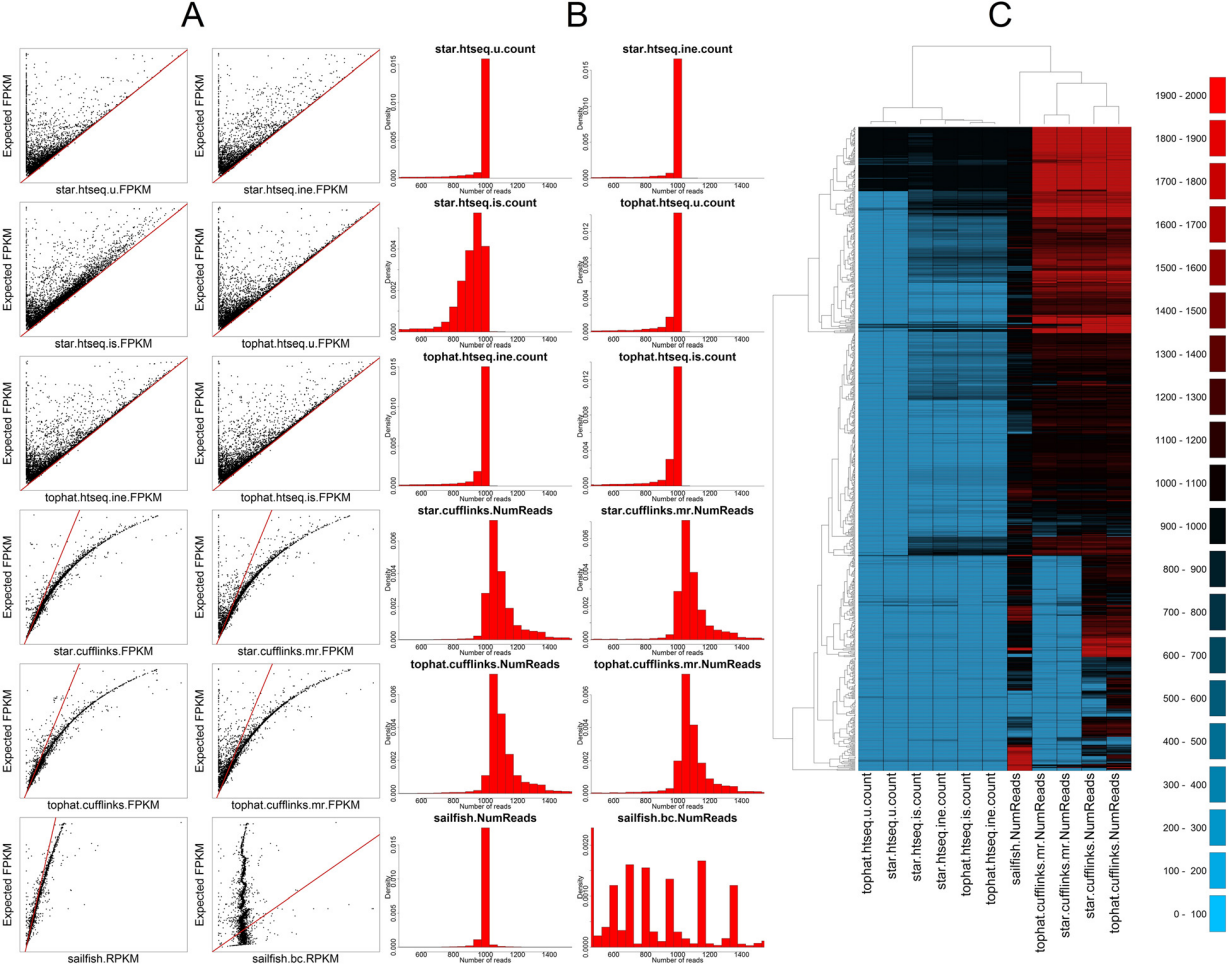
Comparison of methods on global simulated data. **a** Scatter plots comparing FPKM for each of the 12 methods against the known FPKM from simulated data. The *red line* indicates the y = x line. **b** Histograms of read counts for each of the 12 methods. All methods should have a single peak at 1000. **c** A heatmap of read counts from 843 grossly underestimated genes and 187 grossly overestimated genes. *Black* and darker colours indicate read counts close to 1000 (accurate); *green* colours indicate underestimation and *red* colours overestimation

The scatterplots in Fig. 1a show observed versus expected FPKM for all methods. In general, the methods using HTSeq show a linear relationship between observed and expected values, but also provide the most false negatives (where expected FPKM > > observed FPKM). The results from Cufflinks show two distinct trends in all cases: a distinct curved relationship between observed and expected FPKM, and an overall trend to overestimate the FPKM. The overestimation is greater for shorter transcripts. Correcting for multi-mapped reads in Cufflinks increases the number of false negatives. Finally, the Sailfish results show a very linear relationship between observed and expected, albeit with a slight tendency for overestimation. The Sailfish graph is distorted by a single gene whose expression is vastly overestimated — GAGE2E, a member of the GAGE gene family that has been implicated in many types of cancer (see below). The bias-corrected Sailfish results show almost no relationship between observed and expected FPKM.

The barplots in Fig. 1b show histograms of the estimated number of reads assigned to each gene, and we would expect to see a single large peak at 1000. The read counts data confirm the results from the FPKM data — all but one method (Sailfish bias-corrected) has a large peak close to 1000; read counts from HTSeq have a very strong peak at 1000, but tend to show a long tail of false negatives; read counts from Cufflinks have a peak at 1000, but a wider distribution in general and show a tendency to overestimate; Sailfish appears to be very accurate; and the Sailfish bias-correction method hasn’t worked well on these data. Of note is the star.htseq. is.count histogram, which shows a peak below 1000, and a larger number of false negatives. This pattern is not seen in the tophat.htseq. is.count, suggesting that there is an aligner-specific effect of the “intersection strict” parameter in HTSeq.

Finally, the heatmap in Fig. 1c includes all genes where the estimated number of reads is less than 100 (n = 843) or greater than 1900 (n = 187) in at least one method (n = 958). We can see large numbers of genes whose expression is underestimated by HTSeq-based approaches. Whilst some of those genes are accurately measured by Cufflinks-based approaches, others are either over- or underestimated. The overall impression is that none of the methods provide an accurate picture of the expression of all of these genes, with each method showing relatively large numbers of genes with either under- or overestimated read counts.

### Characteristics of problematic genes

**Fig. 2:**
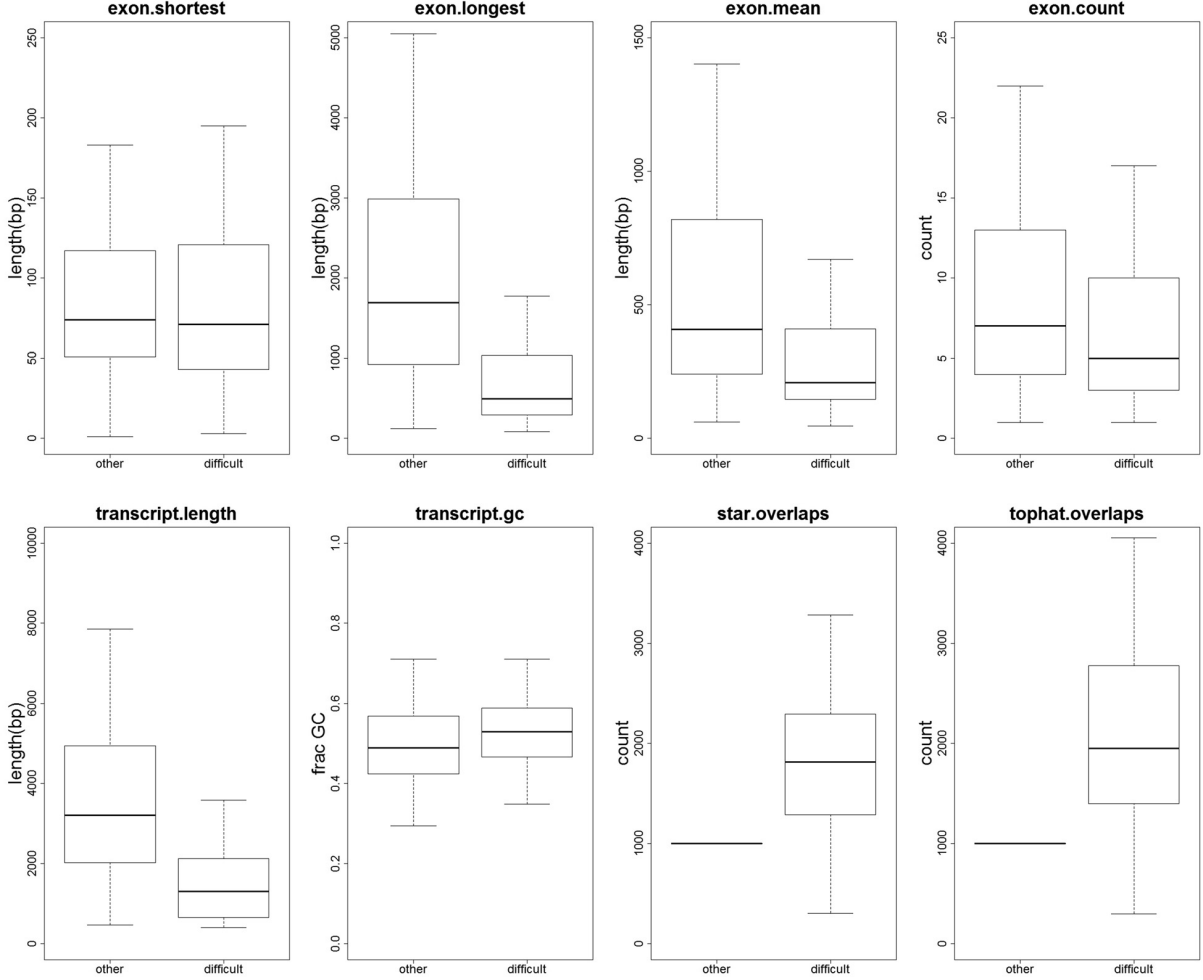
General characteristics of problematic genes. Boxplots comparing the length of the shortest exon, the length of the longest exon, the mean exon length, the total number of exons, the transcript length, transcript percentage GC, the number of reads overlapping from the STAR alignment and the number of reads overlapping the TopHat alignment for the 958 problematic genes and the 18,696 other genes

Having identified 958 problematic genes whose expression is either severely over- or underestimated by at least one method, we looked to see if there were any general characteristics of the problematic genes. Minimum, maximum and mean exon length, total number of exons, transcript length, percentage GC, and the number of reads overlapping the transcripts from both the TopHat and STAR alignments were calculated for all 19,654 genes. Figure 2 shows a boxplot comparing those statistics for the 958 problematic and 18,696 remaining genes.

In the group of problematic genes, the longest exon tended to be shorter, as did the mean exon length. Problematic genes tended to have a slightly lower number of exons, and the transcripts tended to be shorter. There was a very slight tendency for problematic genes to have a higher GC content; however, most striking is the number of reads overlapping problematic genes. On average, problematic genes had 2164 unique fragments overlapping their exons from the STAR alignment, and 2680 unique fragments overlapping their exons from the TopHat alignment. As we simulated 1000 reads from each gene, this indicates that one of the major issues for RNA-Seq quantification is multi-mapped reads and the resolution of mapped fragments to a single gene.

The heatmap in Fig. 1c includes all genes where the estimated number of reads is less than 100 (n = 843) or greater than 1900 (n = 187) in at least one method (n = 958). The heatmap can be broken down into four groups: at the top is a group of genes where the accuracy is high for HTSeq-based approaches and Sailfish, but where Cufflinks overestimates. These genes tend to be very short, with low numbers of exons, and have relatively low numbers of multi-mapped reads (Additional file 2: Figure S1). Below that group is a group of genes where the read count is underestimated by HTSeq, overestimated by Cufflinks, and where Sailfish is accurate. These genes also tend to be short in length, with low numbers of exons, but have high numbers of multi-mapped reads (Additional file 3: Figure S2). The third group is a group of genes where the read count is underestimated by HTSeq, and both Sailfish and Cufflinks approaches are accurate. These genes are of normal length, and have high numbers of multi-mapped reads (Additional file 4: Figure S3). The fourth group is very similar to the third, except the addition of the --multi-read-correct parameter to Cufflinks results in underestimation. This group also contains a group of genes which Sailfish overestimates. These genes again tend to be shorter than normal, with very high numbers of multi-mapped reads (Additional file 5: Figure S4).

### RNA-Seq underestimates expression in genes relevant to human disease

Having identified a set of genes whose gene expression current bioinformatics methods are unable to accurately measure, we wanted to identify and emphasize the importance of those genes in human disease. The full results, including estimates of read counts and FPKM, from all 12 methods for all 19,654 genes can be seen in Additional file 1: Table S1.

The Y-chromosome deleted-in-azoospermia (DAZ) gene family is associated with the AZFc (azoospermia factor c) phenotype of male infertility [9]. The AZFc region of the Y chromosome is highly susceptible to structural variations due to the presence of repetitive amplicons. Four DAZ gene copies are located on the Y chromosome in palindromic duplications and are expressed in the human testis with highly polymorphic expression [10]. Deletion of DAZ genes in humans has been correlated with male infertility in both a South Chinese [11] and a Tunisian population [12]. Similarly, RPS4Y2 lies within the AZFb locus, also suggesting a link with male infertility [13]. RBMY1, part of the RBMY gene family, has been shown to be involved in the regulation of sperm motility [14], and deletion of it has been associated with male infertility [15]. Ten genes in the dataset are annotated as DAZ1 to DAZ4. Expression of all the DAZ genes (DAZ1–DAZ4) is totally missed by the HTSeq methods, with all read counts close to zero. Sailfish reports read counts and RPKM (reads per kilobase per million) close to zero for seven of the ten genes, and overestimates the gene expression by 1.65 to 5.36 times the expected FPKM value for the remaining three. The Cufflinks approaches do reasonably well on four DAZ transcripts, but underestimate in the other six (read counts < 210), and the multi-read-correction parameter results in all transcripts having FPKM and read counts close to zero. Two genes are annotated as RPS4Y2 in our data. HTSeq approaches assign a read count of zero to both members. Tophat.cufflinks and star.cufflinks overestimate the read count (almost three times) and FPKM for one copy, yet assigns values of zero to the second, whereas Sailfish produces accurate estimates for both. There are ten members of the RBMY1 gene family. HTSeq underestimates in all cases; both Cufflinks methods perform variably with slight under- and overestimates. Sailfish overestimates for five of the genes, yet underestimates for the remaining five.

Genes in the cancer/testis (CT) multigene families are expressed in numerous cancer types and members are the targets for cancer immunotherapy [16]. Two classes of CT gene families are defined based on the genomic locations of their gene members: genes are either located on autosomes or located often as clusters of genes on the X chromosome. The CT47A subfamily consists of 13 genes arranged as direct tandem repeats within the Xq24 region of the X chromosome, recently resolved by longer read lengths (36–42 kb) achieved by the MinION Nanopore sequencers [17]. The biomedical relevance of this gene family is further highlighted by the fact that the Xq24 genomic region and the CT47A locus have been reported to contain structural variants associated with X-linked intellectual disability [18] and X-linked mental retardation [19]. A related gene family member, CT45A7, has been reported to be expressed in lung cancer [20]. Twelve genes are annotated as being part of the CT47A gene family in our simulated dataset. HTSeq misses the expression of all the genes in this family; Sailfish strongly underestimates the gene expression of 7 out of the 12 CT47A genes (with a ratio of observed versus expected FPKM values of 0.04 to 0.08), strongly overestimates the gene expression of a further two genes, and reports an FPKM close to the truth for a further three. The tophat.cufflinks method works best here, reporting FPKM values between 42 and 59 where the expected value is 39. However, the use of the --multi-read-correct parameter results in highly inaccurate results. Interestingly, star.cufflinks underestimates the expression of these genes in all cases (FPKM values between 0.26 and 6.8). On further inspection, whilst TopHat reports between 9829 and 11,077 (mulit-mapped) reads overlapping these genes, STAR only reports between 131 and 398.

The GAGE cancer/testis antigens are expressed in a large number of cancers [21–24] and have been shown to have anti-apoptotic characteristics [25]. Consisting of at least 16 genes within tandem repeats, these are likely to be due to replication under positive selection [26]. GAGE antigens are known targets for tumour-specific cytotoxic lymphocytes in melanoma [27]. Twelve genes are annotated as being part of the GAGE family in our simulated data. All read counts are underestimated by more than 50 % by the HTSeq-based methods, except for GAGE1, which is assigned a read count between 825 and 851 depending on the aligner and parameters chosen. Both tophat.cufflinks and star.cufflinks report read counts and FPKM values above zero for these genes, but almost all are underestimates, and tophat.cufflinks performs better than star.cufflinks. Sailfish reports a read count of zero for four of the GAGE genes, underestimates a further six, yet strongly overestimates the final two. One of these, GAGE2E, is the outlier visible in Fig. 1a — assigned over 8000 reads, and with the reported RPKM over five times that of the expected FPKM.

The UTY genes are located within the male-specific region of the Y chromosome (MSY), and Ensembl predicts there are 13 paralogous genes within the group. The genes encode the “ubiquitously transcribed Y chromosome tetratricopeptide repeat protein”. Haplogroup I [28] — a common European lineage of the Y chromosome — is known to be associated with an increased risk of coronary artery disease [29]. This predisposition to coronary artery disease was shown to be associated with the down-regulation of UTY genes (amongst others) in macrophages. Again, 12 genes are annotated as UTY in our simulated dataset. All HTSeq methods underestimate the number of reads in all cases, with most estimates being zero or close to zero. Both star.cufflinks and tophat.cufflinks report FPKM values greater than zero, and both methods under- and overestimate for different members of the family (read counts range from 228 to 2347). Use of the --multi-read-correct parameter did not change the estimates significantly. Sailfish underestimates ten of the members, two with read counts close to zero, and overestimates the final two.

TSPY1 (Testis-specific protein, Y-linked 1) copy number variation impacts on spermatogenetic efficiency and low copy numbers have been associated with infertility in males [30]. The TSPY1 gene is located within the gonadoblastoma locus on the Y chromosome (GBY), and in women presenting abnormal karyotypes the TSPY1 gene is thought to play a major role in gonadoblastoma tumorigenesis [31]. The TSPY gene family has been shown to play a role in testicular germ cell tumours [32] and was proposed as a biomarker for male hepatocellular carcinoma [33]. Additionally, the TSPY gene is expressed in the brain, suggesting a role in neural development [34]. Thirteen genes are annotated as being part of the TSPY gene family in our simulated data. Again, the HTSeq methods grossly underestimate read counts in all cases. Both the star.cufflinks and tophat.cufflinks methods are reasonably accurate, yet this accuracy is removed when using the --multi-read-correct parameter. Sailfish reports a range, from severe underestimation (zero), to overestimation (1.85 times the expected FPKM).

Members of the USP17 family have been linked to apoptosis [35]. NPIPA3 is a member of the NPIP gene family, members of which are expressed in the macula and have been proposed as a susceptibility locus for age-related macular degeneration [36]. TBC1D3C is a member of the TBC1D3 gene family, which has been linked to prostate cancer [37] and tumour formation in mice [38]. DUX4 is a pro-apoptotic protein [39] in the D4Z4 locus, made up of tandem copies of a 3.3-kb repeat, which has been linked to facioscapulohumeral muscular dystrophy [40]. All of the above families display now familiar patterns: HTSeq-based approaches tend to severely underestimate, and star.cufflinks, tophat.cufflinks and Sailfish perform variably, including severe underestimation, severe overestimation or occasionally accurate. In most cases, the use of the --multi-read-correct parameter in the Cufflinks methods decreases accuracy.

It is beyond the scope of this paper to analyse all 958 genes; however, by focusing on the subset above, we have demonstrated that RNA-Seq often underestimates the expression of many genes which are relevant to human disease, and that no single method is accurate in all cases.

### Simulation of reads from problematic genes

Having assigned the same number of reads to each gene in the previous section, we then simulated a second dataset with a variable numbers of reads to test whether we had unfairly biased the results towards a particular method. Using the 958 problematic genes identified above, we simulated a random number of reads between 100 and 100,000 from each gene. In total we simulated 49 million read pairs; in this second dataset, STAR reported 15.37 million read pairs without a unique mapping (31 %) whereas TopHat reported 16.12 million non-unique fragments (32 %).

Full results can be seen in Additional file 6: Table S2. As expected, each method performs significantly worse on the subset of difficult genes than on the global dataset (Table 3). The two Cufflinks methods perform best (*r* = 0.90), though these drop to 0.83 and 0.82 when the --multi-read-correct parameter is chosen. The HTSeq (union) methods perform particularly badly (*r* = 0.58) and the Sailfish bias corrected method again appears not to have worked on these data.

**Table 3:** Method performance on targeted simulated data

Method	Description	Pearson correlation coefficient
Star.htseq. u	STAR, HTSeq (union)	0.58
Star.htseq.ine	STAR, HTSeq (intersection-nonempty)	0.76
Star.htseq. is	STAR, HTSeq (intersection-strict)	0.75
Tophat.htseq. u	TopHat2, HTSeq (union)	0.58
Tophat.htseq.ine	TopHat2, HTSeq (intersection-nonempty)	0.75
Tophat.htseq. is	TopHat2, HTSeq (intersection-strict)	0.75
Star.cufflinks	STAR, Cufflinks	0.90
Star.cufflinks.mr	STAR, Cufflinks (multi-read-correct)	0.83
Tophat.cufflinks	TopHat2, Cufflinks	0.90
Tophat.cufflinks.mr	TopHat2, Cufflinks (multi-read-correct)	0.82
Sailfish	Sailfish (RPKM)	0.85
Sailfish	Sailfish bias-corrected (RPKM)	−0.14

Pearson correlation coefficients between FPKM from each method and the expected FPKM from simulated data for 958 difficult genes

**Fig. 3:**
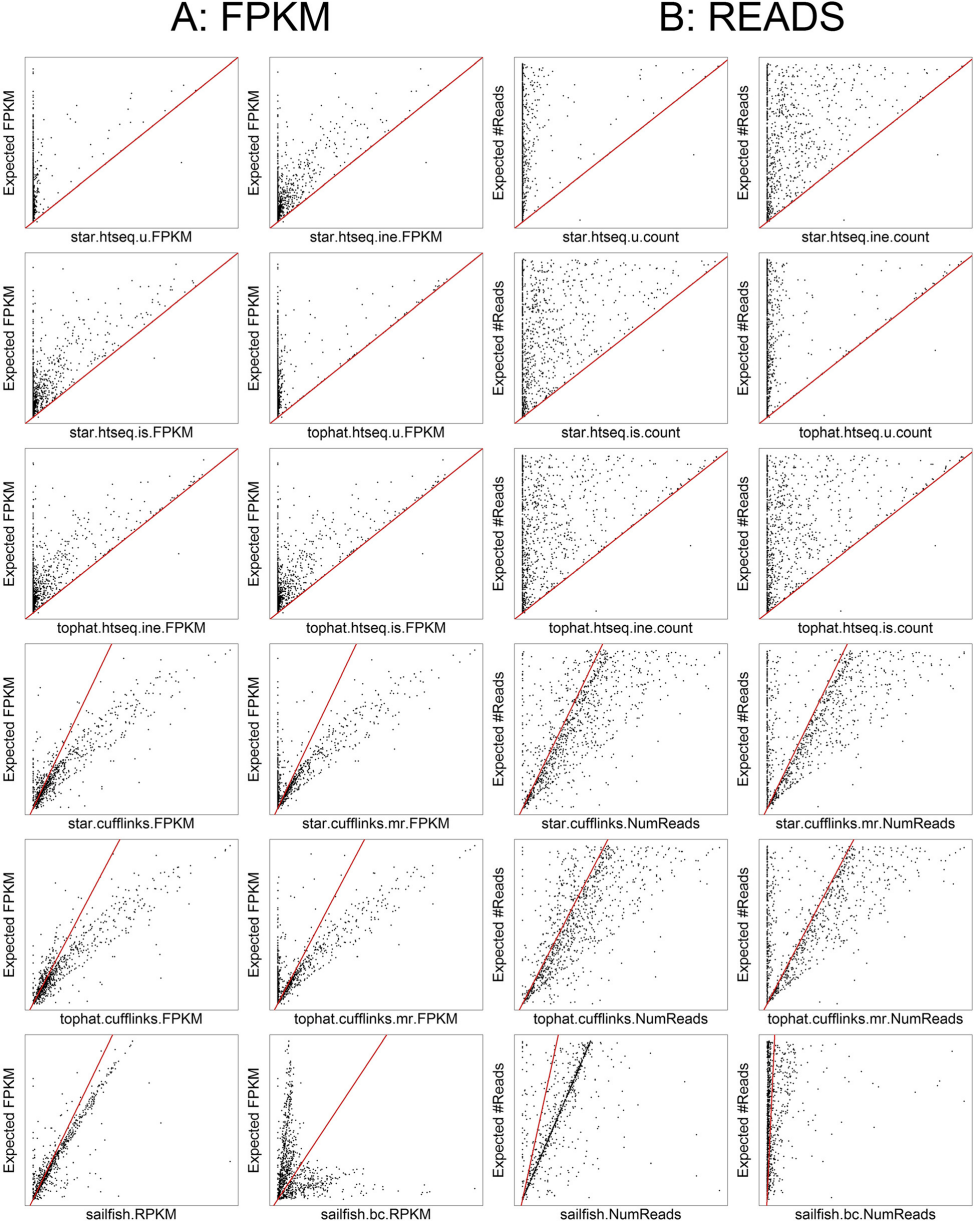
Comparison of methods on difficult genes. **a** Scatter plots comparing observed FPKM for each of the 12 methods against the known FPKM from simulated data. The *red line* indicates the y = x line. **b** Scatter plots comparing observed read counts for each of the 12 methods against the known read counts from simulated data. The *red line* indicates the y = x line

The scatterplots in Fig. 3a show expected versus calculated FPKM values for the 12 methods, and Fig. 3b shows the expected versus observed number of reads. These results reinforce those from the previous section: the HTSeq methods all show large numbers of false negatives; the Cufflinks methods have a tendency to overestimate the FPKM, and the --multi-read-correct parameter increases the number of false negatives; and Sailfish shows a good linear relationship between expected and observed FPKM, albeit with a tendency to overestimate, with a few very-large overestimations.

### Assigning multi-mapped reads to gene-groups reveals biological signal

In our global simulated data, the mapping approaches failed to assign between 2.97 % and 4.33 % of the reads uniquely to a gene. We have used these data to define a list of genes whose expression is difficult to estimate by commonly used methods. Those results are from perfect reads that were simulated from the same reference we use for expression estimation, and we hypothesize that the results would be far worse in real experiments. Many of the problems stem from multi-mapped or ambiguous reads, and there may not be enough information in RNA-Seq data to assign these reads accurately to a single gene.

We therefore propose a two stage analysis: in stage 1, reads are assigned uniquely to genes; and in stage 2, reads that map to multiple genes are assigned uniquely to “multi-map groups” (MMGs). MMGs can be described as groups of genes that multi-mapped reads uniquely map to, consistently across the dataset (see “Materials and methods”). MMGs do not rely on existing annotation, and are derived from the data themselves (see “Materials and methods”).

**Fig. 4:**
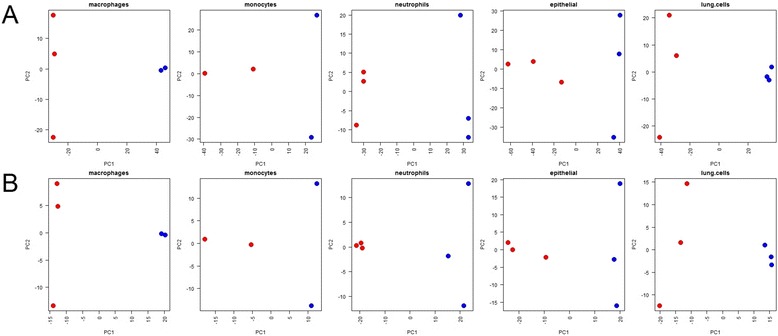
Principle components analysis (PCA) of mouse cancer study. **a** PCA of tumour (*red*) and normal (*blue*) RNA-Seq datasets from each of five cell types. Input data are log(FPKM) values after mapping data using STAR and counting only uniquely mapped reads against known mouse genes (stage 1 analysis) (**b**) PCA of tumour (*red*) and normal (*blue*) RNA-Seq datasets from each of five cell types. Input data are log(FPM) values of reads that cannot be assigned to a single gene but can be uniquely assigned to a multi-map group (MMG). The reads used in (**b**) are only those reads discarded from (**a**)

To demonstrate the efficacy of this approach, we re-analysed five datasets from a recent study of mouse lung cancer [35]. The authors used RNA-Seq and demonstrated cell type-specific differences between the tumour and normal transcriptome in five populations of cells. We re-analysed the data using STAR to align reads to the genome, and HTSeq to count reads that can be uniquely assigned to genes (Additional file 7: Table S3). Using this method, we were unable to assign between 27.8 % and 43.9 % of the reads to a single gene. The major reason for this was the high proportion of multi-mapped reads, although many reads also overlapped either multiple or no features in the annotation. Figure 4a shows the results of a principal components analysis of the resulting FPKM values; despite ignoring between 27.8 % and 43.9 % of the reads, this method is able to reproduce the results from Choi et al. [35]. We can see cell type-specific differences between tumour and normal samples in all five datasets.

We then implemented the stage 2 analysis. Using only reads that otherwise would have been discarded, we assigned all unassigned reads uniquely to an MMG. To reduce noise, only MMGs that had more than 100 reads assigned to them in over 13 of the 27 datasets were kept for further analysis. We collapsed large groups (n ≥ 5) by merging two groups if one was completely contained within the other. Using this method, we were able to “rescue” between 21.6 % and 48.4 % of the discarded reads to 4847 MMGs (Additional file 8: Table S4) containing 5544 genes (genes may be members of more than one MMG). The minimum group size was 1, the maximum 47, and the mean size 3.2. MMGs may be size 1 because sometimes *all* multi-mapped locations overlap the same gene; at other times, one or more mapped regions may be intronic/intergenic, and one or more overlap a gene. In all cases, htseq-count discards the read. In total, 1051 of our MMGs were of size 1, and it would be possible to add these MMGs to the single gene analysis. Of the 4847 MMGs identified, 2431 (50.2 %) contain at least one pseudogene, 4299 contain at least one protein coding gene, and 1402 contain two or more protein coding genes.

Figure 4b shows the results of a principal components analysis on the resulting log FPM (fragments per million) values. We can see that by estimating the expression of MMGs, we can reveal relevant biological signal within the data that would have been discarded by the stage 1 analysis, and we again see cell type-specific differences between tumour and normal samples.

### Differential expression of MMGs

Having accurately and uniquely assigned reads to MMGs, it is now possible to carry out differential expression analysis to identify MMGs that are differentially expressed between tumour and normal samples. Once an MMG has been identified, researchers may use a more targeted technique, such as quantitative PCR, to calculate which genes within the group are differentially expressed.

We first carried out differential expression between tumour and normal lung cells based on the gene counts from unique reads using edgeR [36]. This process identified a total of 5620 differentially expressed genes (Additional file 9: Table S5).

We then carried out an identical analysis on the MMGs, and identified 1541 differentially expressed MMGs between tumour and lung cells, including data on 2292 genes (Additional file 10: Table S6). Of these, 1610 are not found in the list of 5620 differentially expressed genes from the unique counts, indicating that MMG analysis is capable of discovering significant results that might otherwise have been ignored.

**Fig. 5:**
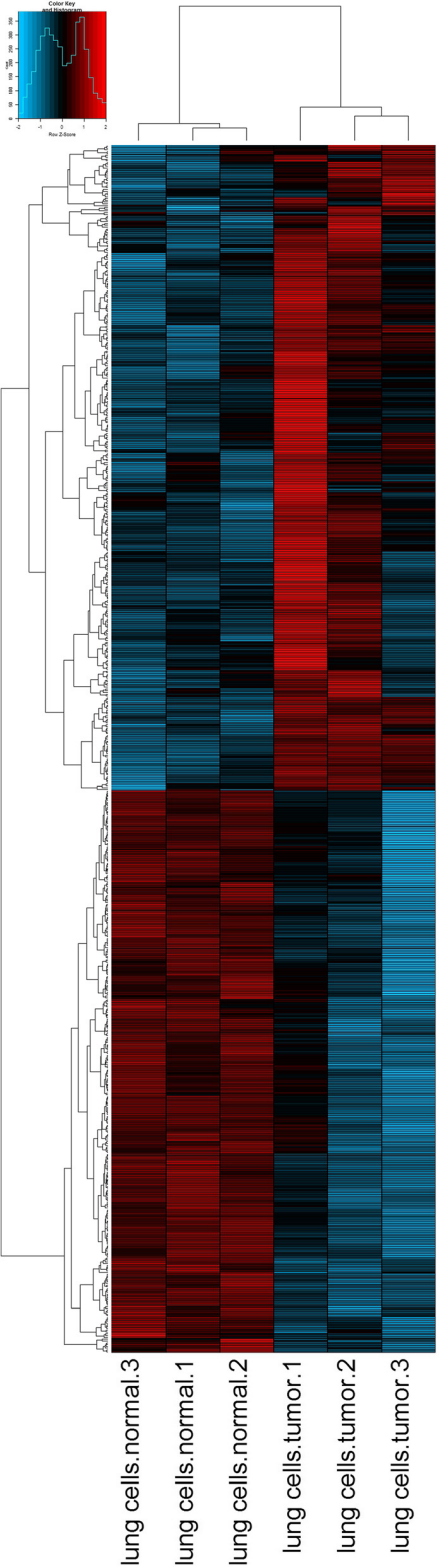
Heatmap of novel multi-map groups (MMGs). A heatmap of the log FPM (fragments per million) values for 672 differentially expressed MMGs that do not contain any genes present in the list of differentially expressed genes from an analysis of unique counts. The heatmap demonstrates that MMGs which are exclusive of differentially expressed genes from unique counts can be used to separate tumour from normal samples

To demonstrate that analysis of MMGs can discover information not present in the analysis of unique counts, we removed all groups from the list of differentially expressed MMGs that contained a member of the 5620 differentially expressed genes from stage 1. This leaves 672 MMGs (Additional file 11: Table S7). A heatmap of the log FPM (fragments per million) values is shown in Fig. 5, and demonstrates that MMGs which are exclusive of differentially expressed genes from unique counts can be used to separate tumour from normal samples.

To highlight further that MMG analysis can discover information not present in the analysis of unique counts, we show data from two relatively highly expressed MMGs (logCPM ≥ 7).

**Fig. 6:**
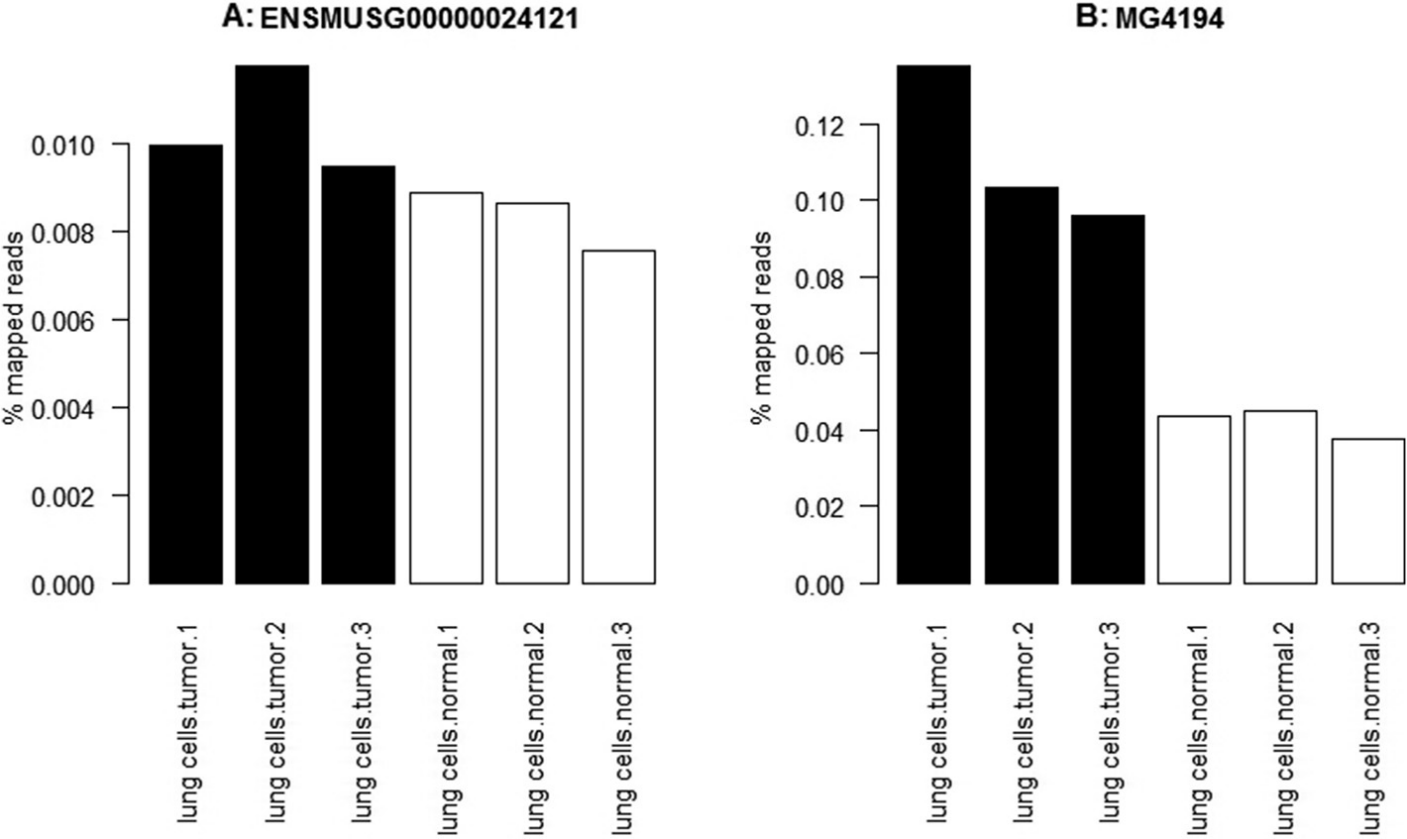
Comparison of read counts for (**a**) ENSMUSG00000024121 and (**b**) MG4194. Read counts expressed as a percentage of the mapped reads for gene ENSMUSG00000024121, and MG4194, a single-gene MMG that contains only ENSMUSG00000024121. ENSMUSG00000024121 was not found to be differentially expressed by the unique read analysis, but MG4194 was found to be differentially expressed by the MMG analysis. *Black bars* represent tumour samples, *white bars* normal samples

MG4194 contains a single gene, “ENSMUSG00000024121”, a protein-coding gene for Atp6v0c (ATPase, H+ transporting, lysosomal V0 subunit C). ENSMUSG00000024121 was not found to be differentially expressed by the unique read analysis, but MG4194 was found to be differentially expressed by the MMG analysis. A comparison of the percentage of mapped reads is shown in Fig. 6, and we see a clear difference between tumour and normal samples in the MMG data. This gene is known to be expressed in the lung [37], and inhibition of ATPases has been shown to reduce the activity of prometastatic proteases [38].

Finally, MG994 contains three genes, Plac9a (ENSMUSG00000095304), Plac9b (ENSMUSG00000072674) and pseudogene Gm9780 (ENSMUSG00000094800). The unique read analysis assigns a read count of zero to all three genes, yet analysis of the MMG shows that there are several thousand reads mapping to the normal cell samples, and several hundred to the tumour cell sample (Table 4). This MMG is reported as differentially expressed by edgeR.

**Table 4:** Gene and MMG counts for MG994

	Lung cells tumour 1	Lung cells tumour 2	Lung cells tumour 3	Lung cells normal 1	Lung cells normal 2	Lung cells normal 3
ENSMUSG00000072674	0	0	0	0	0	0
ENSMUSG00000094800	0	0	0	0	0	0
ENSMUSG00000095304	0	0	0	0	0	0
MG994 (raw counts)	541	721	325	3298	3268	1471
MG994 (percentage total)	0.007	0.009	0.004	0.043	0.037	0.019

Counts data for MG994. The three genes included in MG994 have a read count of zero from the unique read analysis. However, MG994 has many reads that map uniquely within the group, and is differentially expressed between normal and tumour cells

## Discussion and conclusions

We have shown that popular methods used to estimate gene expression from RNA-Seq data often under- or overestimate the expression of hundreds of genes, and many of those are relevant to human disease. We propose a simple but effective method that can be generically applied and which can reveal biological signal in data that would otherwise have been discarded.

Logically, any method which uses sequence similarity to assign reads to a given annotation will struggle when features within that annotation share high sequence similarity themselves. Thus, gene families within the human genome present a problem for RNA-Seq, as bioinformatics methods will find it difficult to determine the correct gene from which a given read originates. The 843 genes assigned a read count less than 100 by at least one of the methods we tested are enriched for paralogues (Fisher’s exact test, *p* = 0.00012), which supports our observation that gene families are problematic. Furthermore, it has been widely reported that human monogenic disorders are enriched for gene duplications [34], which led us to the hypothesis that RNA-Seq may struggle to estimate the expression of many genes of relevance to human health and disease. Indeed this appears to be the case — in this paper we identify hundreds of genes whose expression is grossly underestimated or overestimated by a range of methods, and we describe many of their roles in disease.

The fact that RNA-Seq does not measure all genes accurately is in itself not novel; however, many “unknown unknowns” remain; not only does RNA-Seq not measure accurately the expression of certain genes, but researchers do not know which genes are affected. By publishing the results of 12 commonly used methods on 19,654 human protein-coding genes, we reveal the accuracy of each method for each gene. This is an important resource for researchers carrying out RNA-Seq in humans. Not only can researchers look up lists of genes to see whether they can be accurately estimated, but these data will also help inform the choice of bioinformatics software if researchers know a priori which genes are likely to be involved in their study; alternatively, researchers may choose to run several different pipelines and take a consensus approach.

The purpose of our study is not to criticise the methods themselves — they are all accurate methods for estimating gene expression. However, as sequencing comes closer to the clinic, and with the possibility that sequencing data may be used to inform clinical decisions, it is important to focus not only on what we can measure accurately, but also what we cannot.

The performance of the methods we tested varied, and no single method accurately estimated gene expression in all cases. By simulating reads with zero errors, and using the same reference genome to both simulate and quantify expression, we are giving the tested methods the best possible chance of success. Therefore, any problems we encountered indicate systematic biases in the methods themselves. With data from real experiments, less than perfect data from a transcriptome which is noisier than the reference, the results are likely to be far worse.

The various methods can be classified into model-based and count-based methods. The HTSeq methods are count-based and produce a pleasing linear relationship between expected and observed FPKM for genes with high proportions of uniquely mapped reads. However, by ignoring multi-mapped reads (a deliberate choice [6]), the software produces many more false negatives than other approaches. HTSeq makes no attempt to re-assign multi-mapped reads to the correct gene, and multi-mapped reads are discarded by default. As well as increasing the number of false negatives, it also eliminates false positives. The union, intersection-strict and intersection-non-empty parameters affect how HTSeq deals with uniquely mapped reads and the features they overlap. The intersection-strict method is the most strict, and only assigns reads to a feature if those reads are completely contained within a single feature. Both the union and intersection-non-empty approaches allow partial overlaps with features, and union attempts to resolve reads that overlap two features whereas intersection-non-empty does not. The use of the intersection-strict approach seemed to negatively impact the results from STAR more than the results from TopHat. Inspection of the HTSeq output reveals that the star.htseq. is.count approach had a far higher number of alignments assigned to the group “__no_feature” (1,451,559) than tophat.htseq. is.count (201,658). The “__no_feature” group is used by HTSeq when reads overhang the end of exon features, or overlap introns. The set of genes where tophap.htseq. is.count is accurate and star.htseq. is.count is not includes many genes with very short exons, and it may be that TopHat is slightly better than STAR at aligning reads precisely to short exons. A deeper comparison of these two aligners is required, but is outside the scope of this manuscript.

Globally, the choice of aligner doesn’t appear to have a huge effect on the results, although differences in the parameter settings and algorithms can produce very different results for certain genes, as seen with the number of reads reported for the CT47A genes.

Cufflinks is a model-based approach, and whilst the overall correlation between expected and observed is high in all cases, they do not share a linear relationship. The curved relationship between expected and observed FPKM is due to the “effective length” adjustment performed by Cufflinks. This approach attempts to determine the actual length of transcripts from the data themselves, rather than from the genome annotation. However, we simulated reads from the entire length of transcripts, so annotated length and effective length should be equal. That they are not reveals a potential bias in this approach, which is applied by default, and which can be switched off using the --no-effective-length-correction parameter. The bias more seriously affects shorter genes, and their FPKMs are overestimated. Sailfish is also model-based, and shows the highest correlation between expected and observed. Sailfish RPKM’s show a linear relationship with expected FPKM, albeit with a slight tendency to overestimate. The case of GAGE2E reveals an obvious error in the software. There are other genes where Sailfish vastly overestimates the number of reads (Additional file 1: Table S1), including members of the DAZ, CT47, GAGE and UTY gene families. All of these have very high numbers of multi-mapped reads, and belong to gene families with a high amount of sequence homology. This suggests that there are simply not enough unique kmers within the Sailfish index to accurately measure the expression of these genes. The complete failure of the bias-correction step in Sailfish is likely to be because our simulated data do not fit the assumptions of Sailfish’s bias-correction model.

By avoiding the alignment stage, it may be that methods such as Sailfish have an advantage over Cufflinks and HTSeq. When measuring gene expression against a known annotation, Sailfish is able to consider all reads in the input dataset; however, Cufflinks and HTSeq are only able to consider those reads that map to locations that overlap genes in the annotation file; in other words, Sailfish can access all of the input reads, whereas Cufflinks and HTSeq only access a subset.

In this paper we propose a two-stage analysis of RNA-Seq data whereby those reads that cannot be uniquely assigned to a single gene in stage 1 are instead assigned uniquely to a group of genes (a MMG) in stage 2. The benefit of this approach is that the MMGs can be derived from the data themselves and do not rely on an existing annotation. We make no assumption about the relatedness of genes within each MMG, other than to state that RNA-Seq reads consistently multi-map to all genes within each group across the dataset. In fact, many groups represent known relationships — for example, MG1 consists of genes ENSMUSG00000038646, ENSMUSG00000074826, and ENSMUSG00000094437, which are identified as paralogues within Ensembl. We didn’t use this known paralogous relationship in the analysis — MG1 was derived solely from the multi-mapped reads. Whilst it is not a focus of this paper, finding MMGs in multiple experiments may be a novel way of discovering new relationships between genes.

We demonstrate the effectiveness of the MMG approach using a recently published mouse cancer RNA-Seq dataset, and show that biological signal can be discovered in data that would otherwise have been discarded. Using MMGs, we can accurately measure gene expression at the group level. This means that we can also accurately assess differential expression at the group level. We analysed the mouse lung cell data from Choi et al*.* [35] and show that MMG analysis can reveal information about genes that would be missed when one considers only the uniquely mapped reads. Of the differentially expressed MMGs, 672 contained only genes that were not called as differentially expressed in the first stage analysis. Once differentially expressed groups have been identified, it may then be possible to use a more targeted approach to identify precisely which genes within the group are responsible.

A high number of the MMGs we identified contained at least one pseudogene (2431/4847), which indicates that mapping to pseudogenes is a major source of multi-mapped reads. Whilst it is tempting to dismiss pseudogenes and assign reads instead to their functional counterpart, we cannot be absolutely sure that reads which map to both a functional and a pseudogene come from the functional gene.

The MMG approach complements traditional, gene level-based expression analyses. At present, the only option available to researchers is to measure gene- or transcript-level expression, and as we have shown, for a subset of genes the software tools often get this wrong. RNA-Seq simply does not have the resolution to measure those genes accurately. For many genes whose expression we cannot accurately measure, we *can* accurately measure the expression of a group of genes to which they belong. Furthermore, we can derive those groups directly from the data themselves. One argument against group-level expression studies is that individual genes may be differentially expressed, whilst the group is not. Although this is true, for many genes RNA-Seq does not offer the resolution required for gene-level analysis. Using MMGs, we at least (accurately) recover a significant proportion of the data.

Whilst we use MMGs, the method of assigning reads uniquely to groups of genes is generic, and researchers may choose an existing annotation if they wish — for example, gene families, paralogous groups, protein families, or even gene ontology terms or pathways may be used. As long as reads can be uniquely assigned to a single entity, then expression measurements can be compared reliably across samples. As a count-based approach, this method complements the approach implemented in HTSeq, and it becomes possible to rescue many of the reads that HTSeq ignores.

Multi-mapped or ambiguous reads are a significant problem and researchers should not assume that the bioinformatics methods they use handle these accurately. We show that in a recent study in mice, up to 43 % of the reads could not be uniquely assigned to a single gene. We have tested 12 methods and identified a subset of 958 human genes that are problematic for existing methods. We have identified the role many of these genes play in human disease. Finally, we have proposed a simple but novel way of assigning reads to groups of genes, and show that this can be used to discover biological signal in data that may otherwise have been discarded.

## Materials and methods

### Nomenclature

Throughout this manuscript we use the term read (or reads) to refer to both reads from the same fragment in a paired-end dataset. Therefore, when calculating FPKM (fragments per kilobase per million), we count each read pair only once. Whilst Sailfish reports an RPKM (reads per kilobase per million), our calculations suggest this is in fact an FPKM.

### Simulated data

We wanted to isolate and test the process of RNA-Seq quantification, and separate it from biases introduced by other parts of the RNA-Seq workflow. Therefore, we simulated 1000 perfect RNA-Seq reads from each of 19,654 protein-coding transcripts annotated in Ensembl [41] using wgsim [42]. The reads are 100-bp paired-end, with an insert size of 250 bp and zero errors. The transcripts were chosen as follows: we selected only genes annotated on the core chromosomes of Grch38, and further filtered for protein-coding genes longer than 400 bp in length. For each gene in the set, we chose the single longest transcript. The resulting data are 19,654,000 paired-end reads.

For the targeted simulated data from 958 difficult genes, we simulated a random number of read pairs between 100 and 100,000 for each gene using the same method. The resulting data are 49,431,873 paired-end reads.

### Calculating gene expression and read counts

We tested 12 different RNA-Seq quantification workflows: alignment with STAR [5] or TopHat [4], followed by quantification by htseq-count [6] with each of three options: union, intersection_strict and intersection_empty; alignment with STAR [5] or TopHat [4] followed by quantification with Cufflinks [2], both with and without multi-read correction (−−multi-read-correct); and Sailfish [7], both raw and bias-corrected results (Table 1). Upon inspection of the results, the bias-corrected data from Sailfish were eliminated from further analyses.

Some methods report read counts, some methods report FPKM and others report both. To translate between FPKM and read count, we used the following formulae:

$$ \begin{array}{l}\mathrm{FPKM} = \left({\mathrm{R}}_{\mathrm{c}}/\ \mathrm{T}\right)\ /\ {\mathrm{R}}_{\mathrm{m}}\\ {}{\mathrm{R}}_{\mathrm{c}} = \left(\mathrm{FPKM}\ *\ {\mathrm{R}}_{\mathrm{m}}\right)\ *\ \mathrm{T}\end{array} $$

where R_c_ is the read count assigned to a gene; T is the transcript length in kilobases; and R_m_ is the total number of reads in millions.

### Analysis of mouse lung cancer data with multi-map groups

We propose a simple two-stage RNA-Seq analysis which will help extract information from reads that cannot be assigned to a single gene. In the first stage, reads that can be assigned uniquely to a gene are processed. In the second stage, only those reads that cannot be uniquely assigned to a single gene are analyzed.

We downloaded all data from NCBI Sequence Read Archive (SRA) accession PRJNA256324. In the first stage analysis, reads were mapped to the mouse genome using STAR. We used htseq-count from the HTSeq package to count reads against known genes. Those reads that were ignored by the stage one analysis were used as input to the second stage analysis. The –o option to htseq-count outputs a SAM file describing the fate of each read. For each unassigned read, we compiled a list of all genes the reads mapped to using BEDTools and a series of Perl scripts. These lists of genes formed the basis of the MMGs. Counts against all MMGs discovered in the data across all samples were compiled, and these were then filtered such that only MMGs that had at least 100 reads in at least 13 of the datasets were kept. Large groups (n ≥ 5) were collapsed such that any group that was wholly included within a larger group were merged. All scripts are available from [43].

The heatmap in Fig. 5 was created as follows. Input data were log-transformed fragments-per-million (FPM) values. The Pearson correlation matrix was calculated for MMGs and samples, and converted to a distance matrix by subtracting from 1. The heatmap was drawn using the heatmap.2() function in R, scaling the data by row.

### Differential expression

Differential expression was carried out on raw read counts for genes and MMGs, respectively, using edgeR [36]. Normalisation factors were calculated and applied, and the common dispersion and tag-wise dispersion estimated. We computed gene-wise exact tests to test for differences between the means of tumour (samples SRR1528732, SRR1528733, and SRR1528734) and normal (samples SRR1528735, SRR1528736, and SRR1528737) lung cells based on the negative-binomial distribution. *P* values were adjusted for the false discovery rate.

## Additional files

Additional file 1: Table S1. Counts and FPKM for simulated data from all genes. FPKM and read counts calculated by 12 different methods for 19,654 human protein-coding genes. Can be downloaded from [43]. (XLSX 7248 kb) Additional file 2: Figure S1. General characteristics of first problematic group. Boxplots comparing the length of the shortest exon, the length of the longest exon, the mean exon length, the total number of exons, the transcript length, transcript percentage GC, the number of reads overlapping from the STAR alignment and the number of reads overlapping the TopHat alignment for a group of genes where HTSeq and Sailfish are accurate, but Cufflinks overestimates. (JPEG 569 kb) Additional file 3: Figure S2. General characteristics of second problematic group. Boxplots comparing the length of the shortest exon, the length of the longest exon, the mean exon length, the total number of exons, the transcript length, transcript percentage GC, the number of reads overlapping from the STAR alignment and the number of reads overlapping the TopHat alignment for a group of genes where HTSeq underestimates, Cufflinks overestimates and Sailfish is accurate. (JPEG 581 kb) Additional file 4: Figure S3. General characteristics of third problematic group. Boxplots comparing the length of the shortest exon, the length of the longest exon, the mean exon length, the total number of exons, the transcript length, transcript percentage GC, the number of reads overlapping from the STAR alignment and the number of reads overlapping the TopHat alignment for a group of genes where HTSeq underestimates, Cufflinks and Sailfish are accurate. (JPEG 576 kb) Additional file 5: Figure S4. General characteristics of third problematic group. Boxplots comparing the length of the shortest exon, the length of the longest exon, the mean exon length, the total number of exons, the transcript length, transcript percentage GC, the number of reads overlapping from the STAR alignment and the number of reads overlapping the TopHat alignment for a group of genes where HTSeq underestimates, Cufflinks and Sailfish are accurate but the use of the --multi-read-correct parameter in Cuffinks results in underestimation. (JPEG 575 kb) Additional file 6: Table S2. Counts and FPKM for simulated data from 958 problematic genes. FPKM and read counts calculated by 12 different methods for 958 problematic human protein-coding genes. Can be downloaded from [43]. (XLSX 311 kb) Additional file 7: Table S3. Unique counts from Choi et al*.* [35]*.* Counts of reads from Choi et al. that map uniquely to genes using STAR and HTSeq. Can be downloaded from [43]. (XLSX 5833 kb) Additional file 8: Table S4. Counts of multi-map groups (MMGs) from Choi et al. [35]*.* Counts of reads from Choi et al. that map uniquely to MMGs. Can be downloaded from [43]. (XLSX 876 kb) Additional file 9: Table S5. EdgeR results from unique counts. Differential expression results calculated by edgeR for gene counts produced by the stage 1 analysis. Can be downloaded from [43]. (XLSX 2159 kb) Additional file 10: Table S6. EdgeR results from MMGs. Differential expression results calculated by edgeR for MMG counts produced by the stage 2 analysis. Can be downloaded from [43]. (XLSX 428 kb) Additional file 11: Table S7. Novel differentially expressed MMGs. The 672 differentially expressed MMGs that do not contain any genes identified as differentially expressed by the stage 1 analysis. Can be downloaded from [43]. (XLSX 67 kb)

## Abbreviations

bp: base pair
CPM: counts per million
CT: cancer/testis
FPKM: fragments per kilobase per million
MMG: multi-map group
RPKM: reads per kilobase per million
