# Supplementary figures and images for: Errors in RNA-Seq quantification affect genes of relevance to human disease
Source: Genome Biol. 2015 Sep 3;16(1):177. doi: 10.1186/s13059-015-0734-x (PMC4558956; doi:10.1186/s13059-015-0734-x)

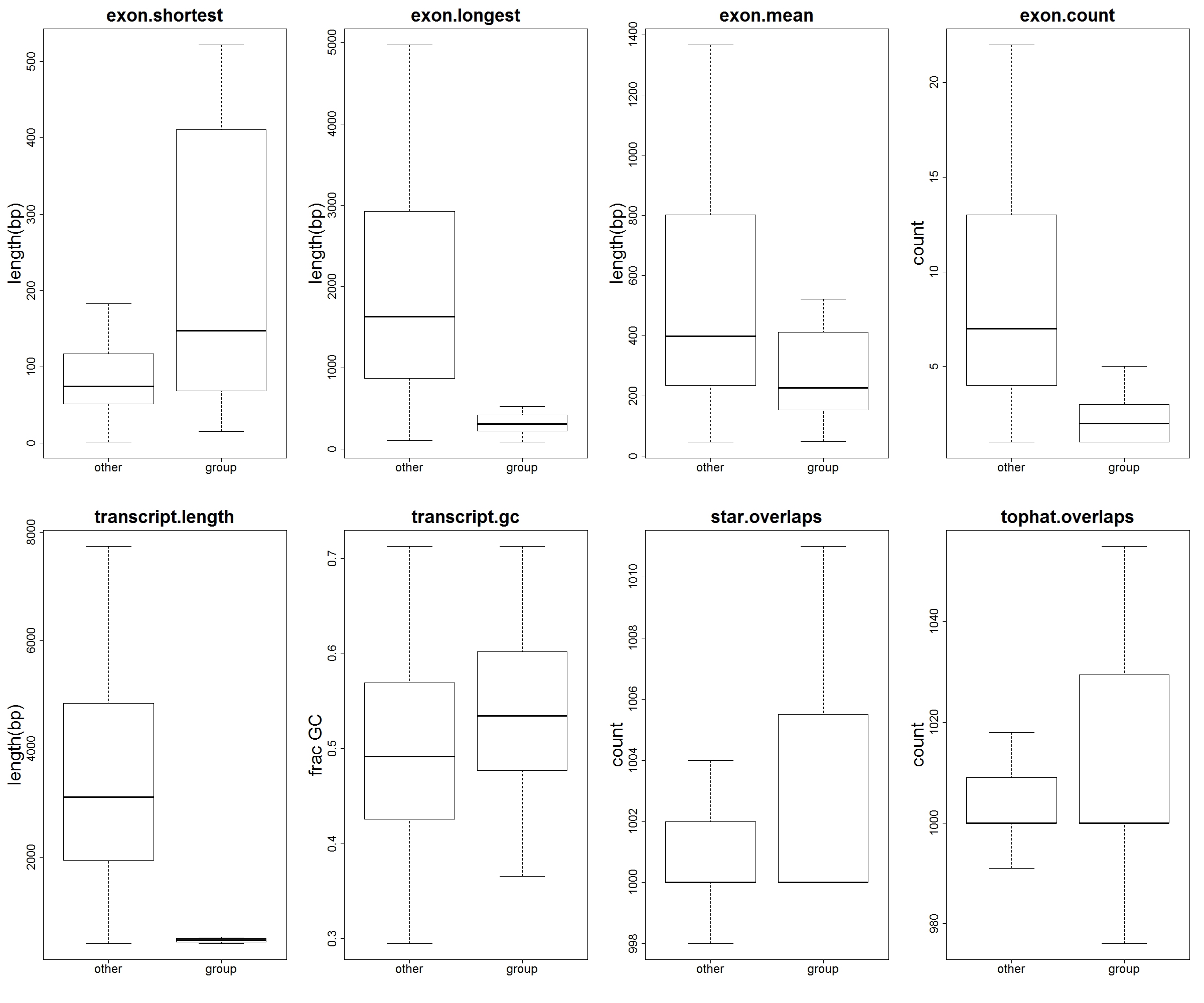

Supplement: Additional file 2: Figure S1. — General characteristics of first problematic group. Boxplots comparing the length of the shortest exon, the length of the longest exon, the mean exon length, the total number of exons, the transcript length, transcript percentage GC, the number of reads overlapping from the STAR alignment and the number of reads overlapping the TopHat alignment for a group of genes where HTSeq and Sailfish are accurate, but Cufflinks overestimates. (JPEG 569 kb) [file 13059_2015_734_MOESM2_ESM.jpg]

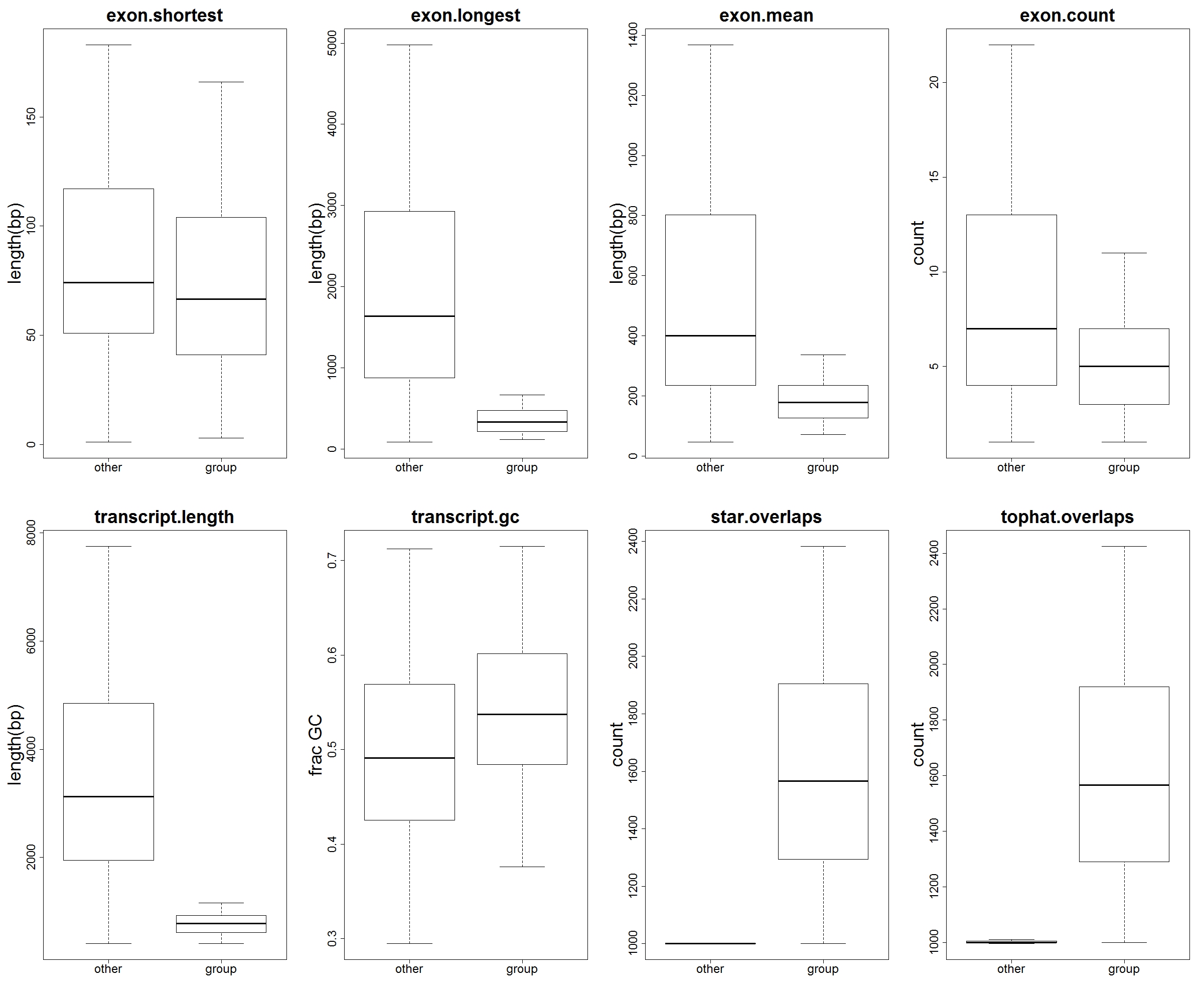

Supplement: Additional file 3: Figure S2. — General characteristics of second problematic group. Boxplots comparing the length of the shortest exon, the length of the longest exon, the mean exon length, the total number of exons, the transcript length, transcript percentage GC, the number of reads overlapping from the STAR alignment and the number of reads overlapping the TopHat alignment for a group of genes where HTSeq underestimates, Cufflinks overestimates and Sailfish is accurate. (JPEG 581 kb) [file 13059_2015_734_MOESM3_ESM.jpg]

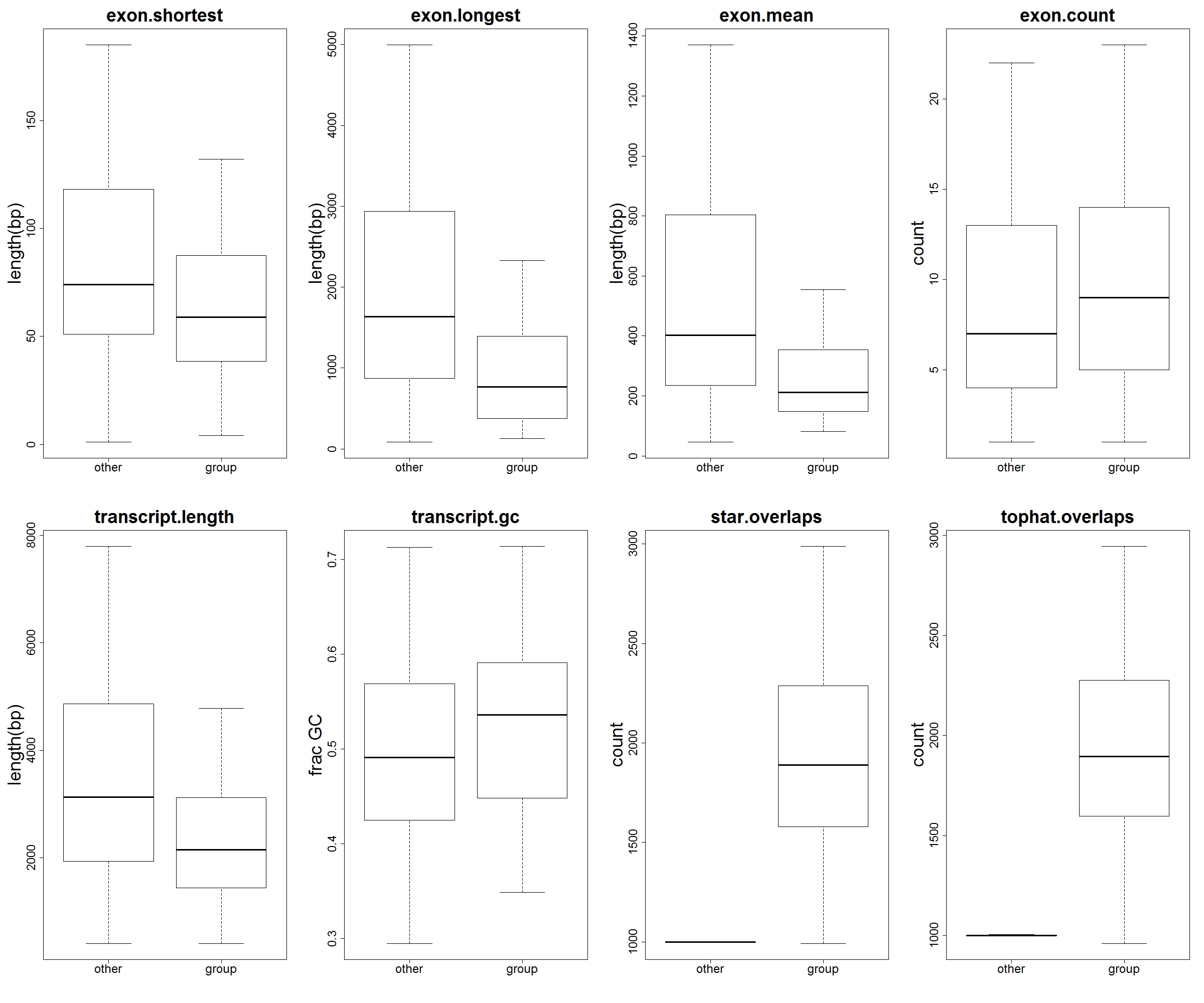

Supplement: Additional file 4: Figure S3. — General characteristics of third problematic group. Boxplots comparing the length of the shortest exon, the length of the longest exon, the mean exon length, the total number of exons, the transcript length, transcript percentage GC, the number of reads overlapping from the STAR alignment and the number of reads overlapping the TopHat alignment for a group of genes where HTSeq underestimates, Cufflinks and Sailfish are accurate. (JPEG 576 kb) [file 13059_2015_734_MOESM4_ESM.jpg]

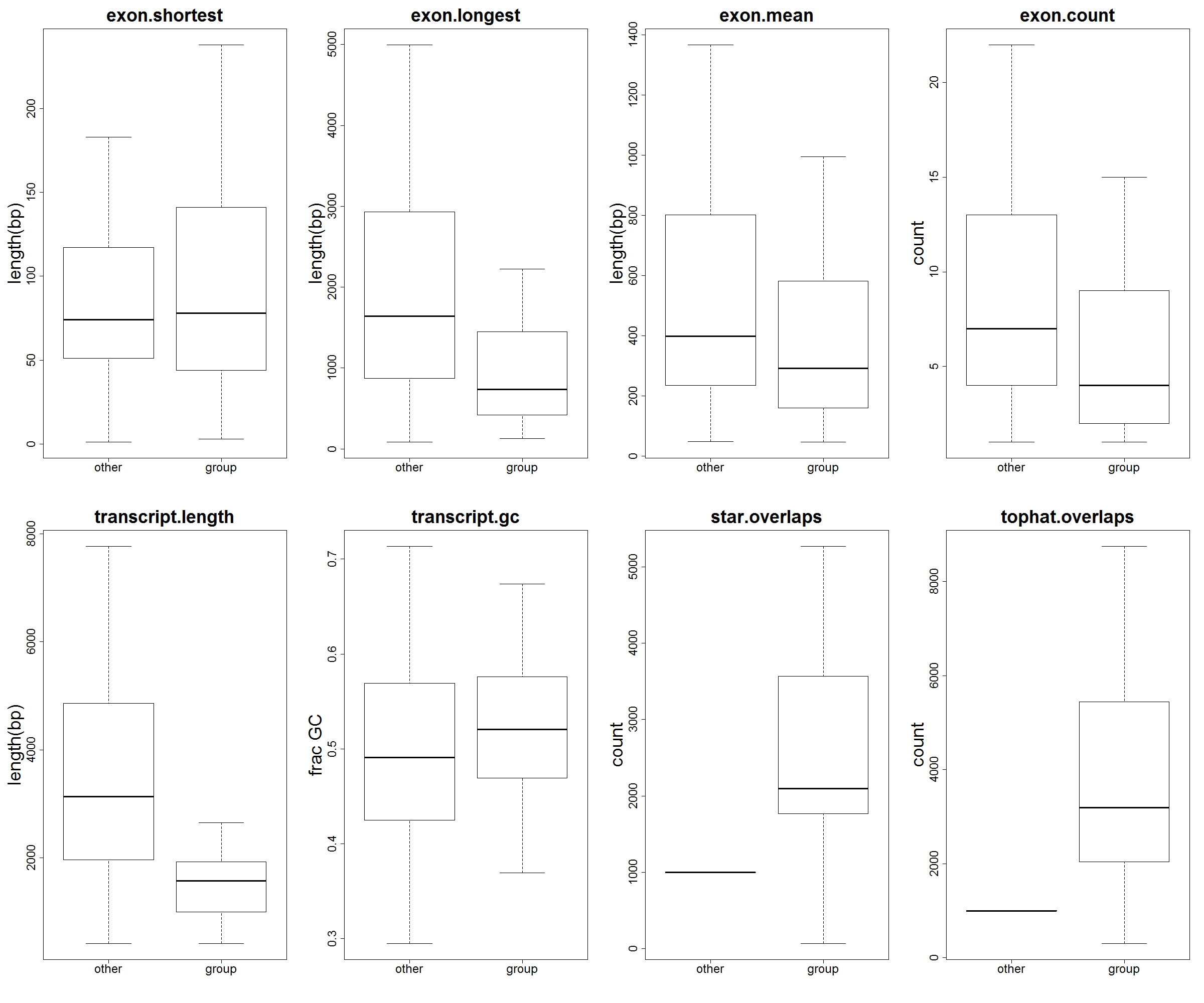

Supplement: Additional file 5: Figure S4. — General characteristics of third problematic group. Boxplots comparing the length of the shortest exon, the length of the longest exon, the mean exon length, the total number of exons, the transcript length, transcript percentage GC, the number of reads overlapping from the STAR alignment and the number of reads overlapping the TopHat alignment for a group of genes where HTSeq underestimates, Cufflinks and Sailfish are accurate but the use of the --multi-read-correct parameter in Cuffinks results in underestimation. (JPEG 575 kb) [file 13059_2015_734_MOESM5_ESM.jpg]
